# Cultivation and morphology of jujube (*Ziziphus Jujuba* Mill.) in the Qi River Basin of Northern China during the Neolithic Period

**DOI:** 10.1038/s41598-024-52260-8

**Published:** 2024-01-27

**Authors:** Yanpeng Li, Xinying Zhou, Keliang Zhao, Junchi Liu, Guanhan Chen, Yaping Zhang, Jiacheng Ma, Nan Sun, Xiaoqiang Li

**Affiliations:** 1 School of Earth Science and Resources, Chang’an University, Xi’an, 710054 China; 2 Key Laboratory of Vertebrate Evolution and Human Origin of Chinese Academy of Sciences, Institute of Vertebrate Paleontology and Paleoanthropology, Chinese Academy of Sciences, Beijing, 100044 China; 3 CAS Center for Excellence in Life and Paleoenvironment, Beijing, 100044 China; 4 University of the Chinese Academy of Sciences, Beijing, 100049 China

**Keywords:** Plant sciences, Ecology, Environmental sciences

## Abstract

This transition from gathering to cultivation is a significant aspect of studying early agricultural practices. Fruit trees are an essential component of food resources and have played a vital role in both ancient and modern agricultural production systems. The jujube (*Ziziphus jujuba* Mill.), with its long history of cultivation in northern China, holds great importance in uncovering the diet of prehistoric humans and understanding the origins of Chinese agricultural civilization. This paper focuses on the domestication of jujube by analyzing the morphology of jujube stones found in three Neolithic sites in northern China's Qi River basin, Zhujia, Wangzhuang, and Dalaidian. The measurements of these jujube kernels are compared with those found in other areas of northern China, as well as modern jujube kernels that were collected. The measurements revealed that the length-to-diameter (L/D) ratio of sour jujube kernels ranged from 1.36 to 1.78, whereas the L/D ratio of cultivated jujube stones varied between 1.96 and 4.23. Furthermore, jujube stones obtained from Zhujia and Wangzhuang sites exhibit pointed ends and possess an elongated oval or narrow oval shape overall, which is indicative of clearly artificial domestication traits. Therefore, this study suggests that jujube was selected and cultivated as an important food supplement in the Qi River basin no later than around 6200 BP.

## Introduction

The transition from wild plant systems to artificially cultivated systems during the Holocene is a crucial event in the history of human and Earth ecosystems^1–3^. Fruit trees, as important food resources, provide humans with essential nutrients, such as proteins, amino acids, and vitamins, and are integrated into traditional and modern agroecosystems worldwide, playing a vital role in human society^4–8^.

To gain a better understanding of the history of domestication, interdisciplinary studies involving archaeology, genetics, and anthropology will be crucial. By combining multiple lines of evidence, researchers can establish a more comprehensive picture of how and when plant domestication took place^1,9,10^. While there is increasing evidence of fruit tree cultivation in the early Holocene, there is still limited knowledge about their evolutionary history^11–15^. Additionally, the relationship between human subsistence strategies and fruit tree utilization, as well as the impact of fruit tree utilization on human society, have been long overlooked. The middle and lower Yellow River basin in northern China is core area of millet agriculture origin and early fruit tree cultivation area^19–21^. Extensive research has been conducted on cereal agriculture in this region, but there is limited understanding of fruit tree collection, utilization process, cultivation history and exchange by prehistoric inhabitants.

Jujube (*Ziziphus jujuba* Mill.), a member of the genus Ziziphhus (*Ziziphus* Mill.) in the family Rhamnaceae, is now widely cultivated in northern China, Central Asia, West Asia and northern Africa^22^. The Jujube fruit is rich in nutrients, has a wide range of medicinal and economic values. It is believed to originated and has a long history of cultivation in the middle and lower reaches of the Yellow River in northern China^23–25^.There are clear records about the cultivation, management, and processing of jujube in ancient literature such as the *Shijing* and the *Erya*, as early as 3000 years ago, which shows that it played a crucial position in the formation of China's dietary culture^23^.

**Figure 1:**
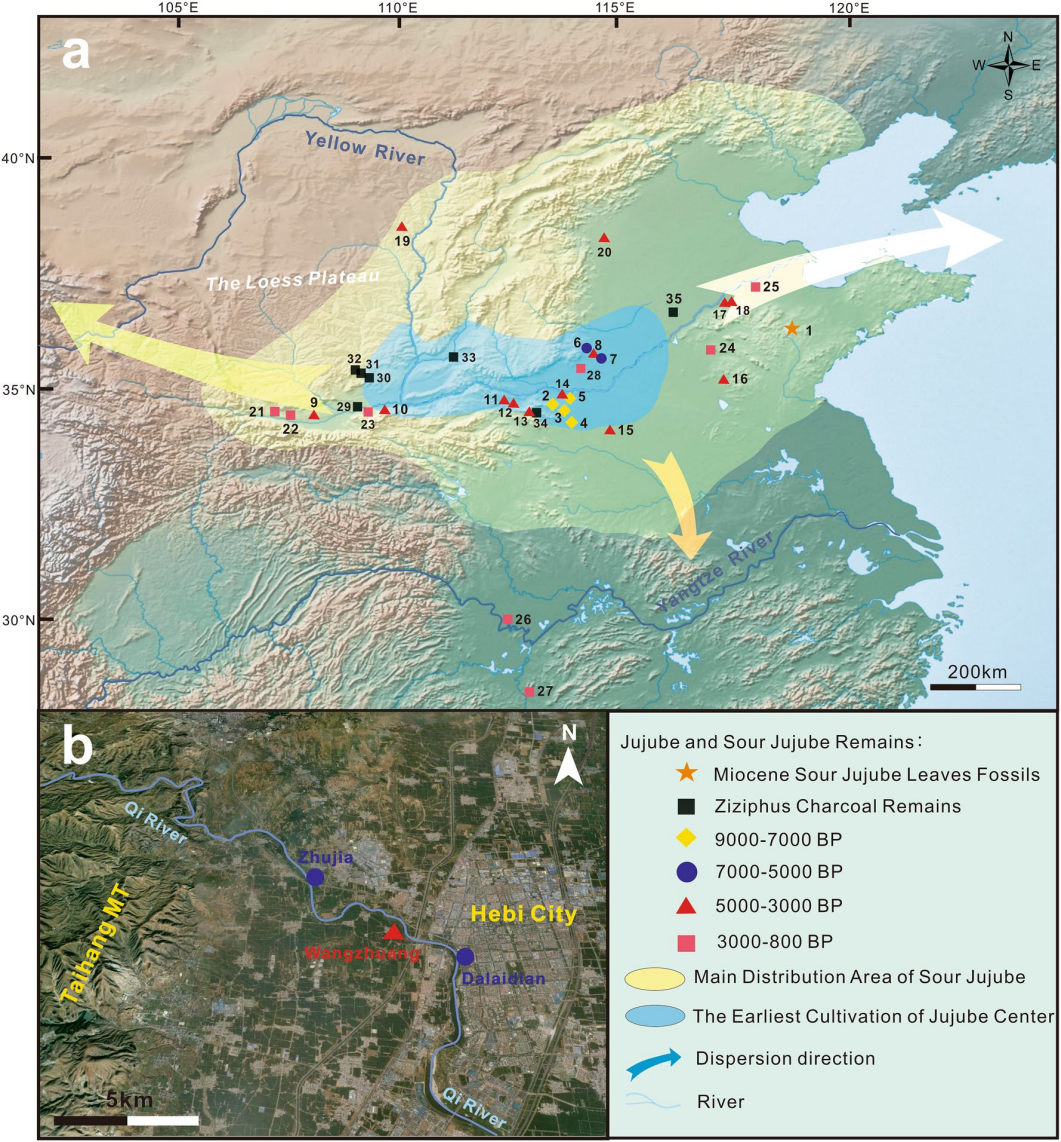
Distribution of Jujube and Sour Jujube Remains in Northern China’s Middle and Lower Yellow River and Overview of the Qi River Basin. (**a**) Distribution of site locations and range of distribution in different periods of the jujube tree. 1.Miocene fossilized sour jujube leaves; 2.Egoubeigang; 3.Peiligang; 4.Shigu; 5.Shawoli; 6.Zhujia; 7.Dalaidian; 8.Wangzhuang; 9.Anban; 10.Nansha; 11.Zaojiaoshu; 12.Wanggedang; 13.Nanwa; 14.Dongzhao; 15.Taomugang; 16.Zhuanglixi; 17.Liujiazhuang; 18.Daxinzhuang; 19.Zhaimaoliang; 20.Taixi; 21.Nanyangsi; 22.Xipu; 23.Koujia; 24.Xiwusi; 25.Chenzhuang; 26.JianglingHan tomb; 27.MawangduiHan tomb; 28.Shangcun; 29.Baijia; 30.Xiahe; 31.Mapo; 32.Nanshantou; 33.Taosi; 34.Wangchenggang; 35.Jiaochangpu. (**b**) Distribution of site locations in the Qi River Basin. This map is created using ArcGIS v10.6 (https://www.esri.com/), in-map labels were added in CorelDraw X8 v18.1.0.690 (https://www.coreldraw.com/). Figure (**a**) base map from (https://www.simplemappr.net/), Figure (**b**) base map from (https://earth.google.com/). See Supplementary Table S1.

Extensive research in the fields of molecular biology has been conducted in recent years, providing compelling evidence on the origin and domestication of jujube, and suggested that jujube originated in the middle reaches of the Yellow River ^25–28^. However, currently, there is a lack of conclusive evidence to support this statement in the context of archaeobotany. While some researchers have discovered charred remnants of Jujube at various archaeological sites, it remains uncertain whether these are cultivated or wild varieties^29^. This ambiguity stems from previous studies lacking a distinct criterion to differentiate between the wild and domesticated types. The main objective of this study is to investigate the cultivation history of jujube and its significance in the prehistoric subsistence. To achieve this, newly discovered charred jujube stones from the Qi River basin, as well as jujube and sour jujube stones gathered from various sites in northern China, were examined (Fig. 1). The findings of this research offer novel evidence regarding the early cultivation, and probably representational morphological evolution of jujube in the northern regions of Neolithic China.

## Results

### AMS 14C dating

A total of four samples were selected for ^14^C dating at the Dalaidian, Zhujia, and Wangzhuang sites. Since kernels were relatively rare at the sites, we selected charred millet seeds and charcoal from the same cultural layer as the kernels for AMS ^14^C dating. Two dating samples were selected from the Zhujia site, including a charred millet seed sample from T3-1 and a charcoal sample from T2-6. At Dalaidian, a charcoal sample was selected for ^14^C dating at 270 cm in the first site 1–1. A total of one dating sample from ash pits 2–4 was selected from the Wangzhuang site. Chronological data for the other sites were obtained from previous studies by our team and published data from others, as detailed in (Table 1).

**Table 1:** AMS^14^C dates for the 13 archaeological sites discussed in this study.

Site	Sample ID	Lab code	Sample type	^14^C age (BP)	Calibrated age (calBP)(95.4%)
Zhujia	ZJ T3-1	Beta-600930	charred seed	5350 + /− 30 BP	6273–6002
Zhujia	ZJ T2-6	Beta -600,929	charcoal	5400 + /− 30 BP	6289–6020
Dalaidian	DLD 1–1 270 cm	Beta -599,783	charcoal	5110 + /− 30 BP	5927–5750
Wangzhuang	WZ 2–4	Beta -600,928	Charred seed	3250 + /− 30 BP	3560–3390
Xiwusi	XWS-1	QZQ806	Charcoal	2435 + /− 40 BP	2703–2354
koujia	KJ-W	OZM466	Wheat	1505 ± 30 BP	1509–1308
Taomugang	TMG-1	QZQ793	Charcoal	3625 + /− 35 BP	4080–3839
Nansha^30^	NS11	OZM459	Charred seed	3279 + /− 31 BP	3390–3560

### Plant macrofossils

The flotation technique was employed to excavate abundant remains from the layer. This study focuses specifically on the jujube and sour jujube kernels that were excavated. Initially, in order to more clearly determine the types of jujube and sour jujube, we first compared and measured the morphology of the modern kernels collected to establish a clear criterion for judging the species. The cultivated jujube kernel is long and slender overall, with pointed ends and a fine fusiform shape, but the differences between varieties are also more disparate. By contrast, the modern sour jujube has a blunt shape at both ends and an ovate or oval overall morphology. We conducted measurements on the length and diameter of the jujube kernel. Our analysis revealed that L/D values of modern sour jujube grown in the wild ranged from 1.19 to 1.68, while those of modern cultivated jujube ranged from 2.28 to 6.37, with obvious distributional boundaries between the two, and the differences in morphology were very significant. Further analysis using a threshold of 1.9 led to the identification of jujube and sour jujube types (see Supplementary Table S2).

Based on the findings of contemporary kernels, we analyzed and measured the morphology of fifteen kernels that were unearthed from the sites. Firstly, the general morphology shows that all the kernels belong to jujube or sour jujube. The kernels excavated from the sites were classified according to the measurement results, and the L/D of sour jujube ranged from 1.36 to 1.78, and that of cultivated jujube ranged from 1.96 to 4.23, which is similar to the distribution trend of modern kernels.

**Figure 2:**
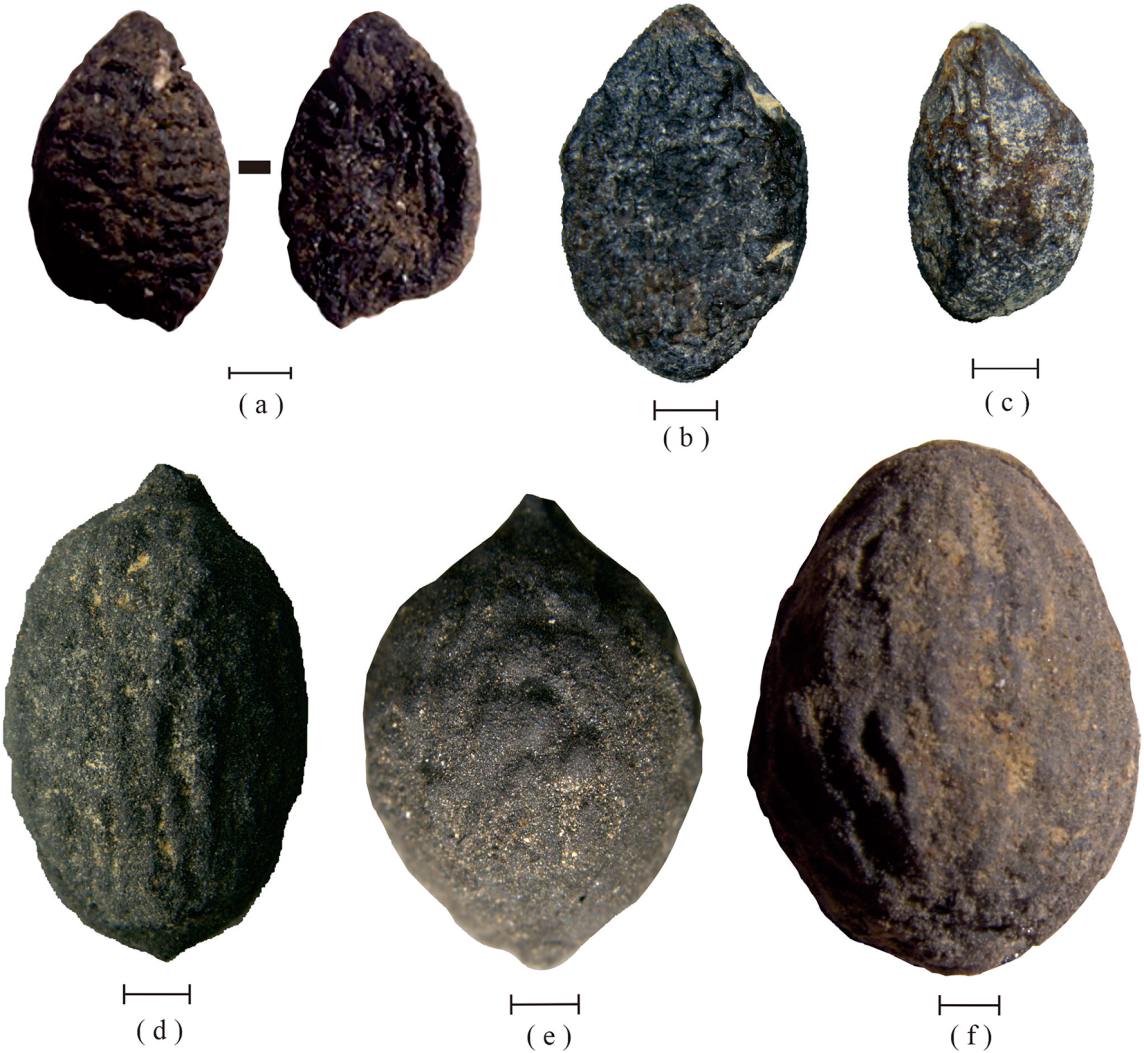
Sour jujube (*Ziziphus jujuba* var. *spinosa*) kernels from the sites. (**a**) Zhujia. (**b**, **c**) Nansha. (**d**) Xipu. (**e**) Xiwusi. (**f**) Dalaidian. Scale bar = 1 mm.

**Figure 3:**
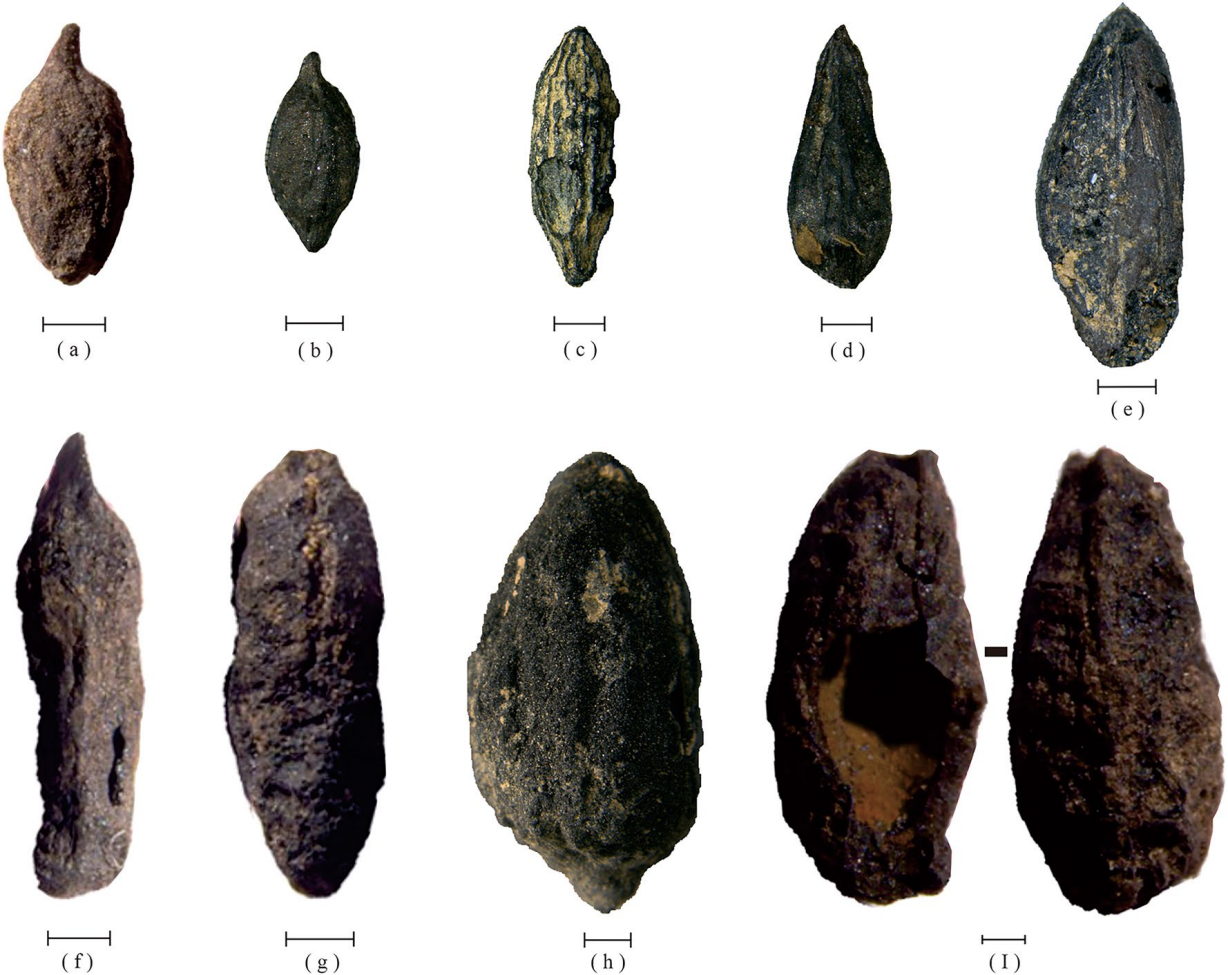
Jujube (*Ziziphus jujuba* Mill*.*) kernels from the sites. (**a**) Wangzhuang. (**b**, **d**) Taomugang. (**c**) Nanyangsi. (**e**) Nansha. (**f**, **g**, **i**) Zhujia. (**h**) Koujia. Scale bar = 1 mm.

Jujube kernels exhibit distinct morphological differences across various sites. Three jujube kernels were excavated from the Zhujia site, each having a long oval or narrow ovate shape with a pointed apex and L/D values of 4.23, 2.75, and 2.05 respectively. The cultivated kernels display a slender overall shape, indicating a clear sign of artificial domestication. In contrast, the remaining kernel (Fig. 2a) has an elliptical shape and an L/D value of 1.59, which implies that it is likely from the seed of a sour jujube. One sour jujube, located at the Dalaidian site (Fig. 2f), exhibits an L/D value of 1.36, possesses an oval shape, and features a blunt apex. Conversely, a cultivated jujube from the Wangzhuang site (Fig. 3a) displays an L/D value of 2.00 and is smaller in size. Fusiform in shape, it presents a prominent, slightly curved tip and a blunt base. Two sour jujubes harvested from the Nansha site (Fig. 2b, c) exhibit L/D values of 1.52 and 1.62, respectively, and possess a rhombus-shaped morphology with a bluntly rounded base and a slightly pointed tip. The remaining sample (Fig. 3e), which is a cultivated jujube, displays an L/D value of 2.45 and has an overall elongated, narrowly ovate structure. The two sour jujubes from Xipu and Xiwusi, depicted in Fig. 2b,c, have L/D values of 1.62 and 1.40, respectively, and are roughly elliptical in shape. Moreover, two cultivated jujubes that were unearthed from the Taomugang site exhibit L/D values of 2.51 and 2.11, respectively. One of these jujubes, shown in (Fig. 3b), is obovate, pointed at both ends, with a long beak at the tip, and the other, shown in (Fig. 3d), is angular-ovate, with an obtuse base and a slightly pointed tip. A jujube that was cultivated at the Nanyangsi location (Fig. 3c) exhibits an L/D value of 2.83, is narrowly elliptical, pointed at both ends, and features noticeable longitudinal grooves. Another cultivated jujube from the Koujia location (Fig. 3h) has an L/D value of 1.96, is fusiform in shape, has a small tip at the bottom, and closely resembles the pome of certain present-day cultivated jujubes.

### The internal structure of the fruit kernels

**Figure 4:**
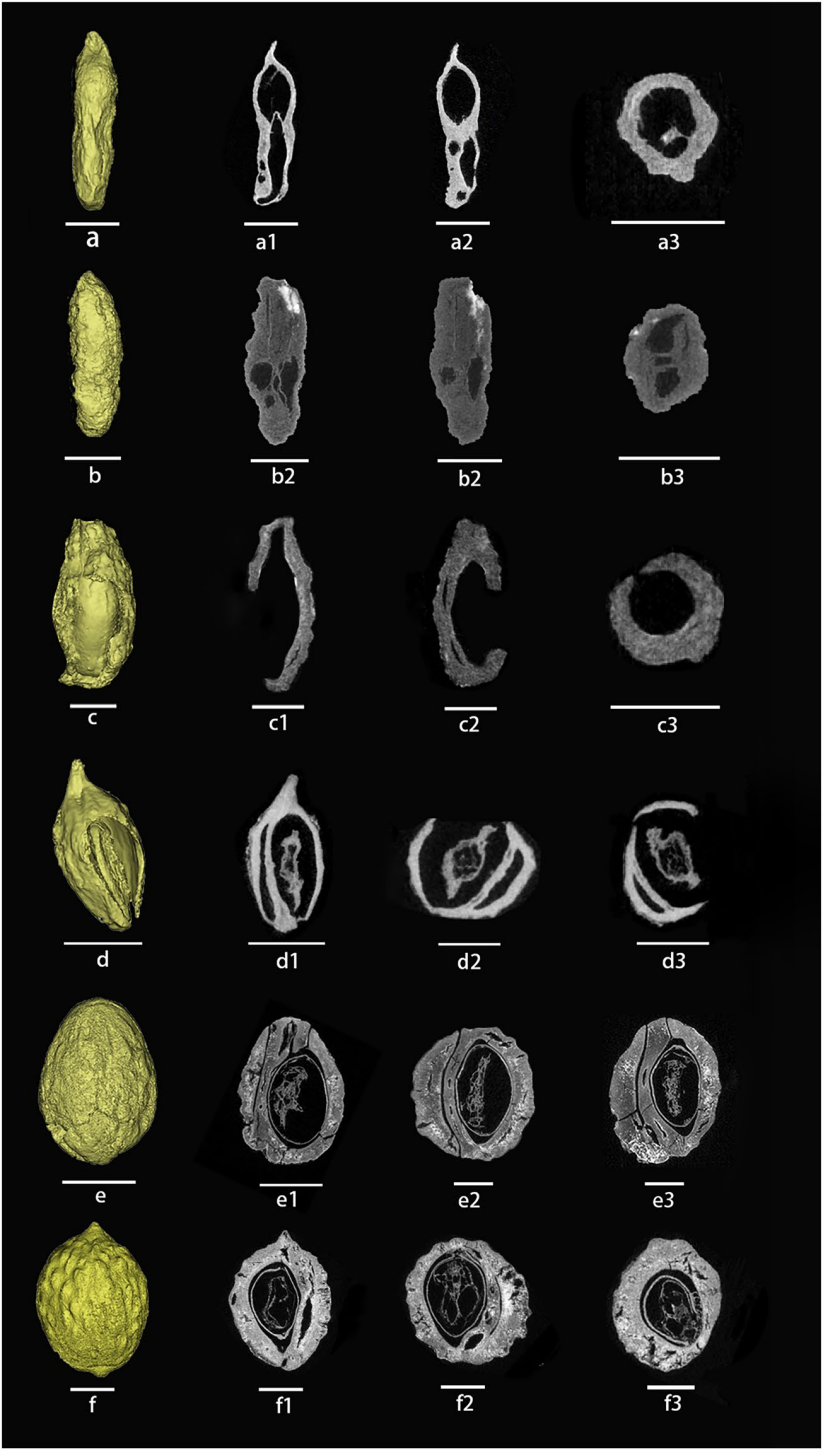
Three-dimensional structures were reconstructed using CT and the internal structure of jujube (**a**–**d**) and sour jujube (**e**, **f**) in different views of the sites. (**a**–**c**) Zhujia; (**d**) Wangzhuang; (**e**) Dalaidian; (f)Xiwusi. Scare bar = 2 mm.

**Figure 5:**
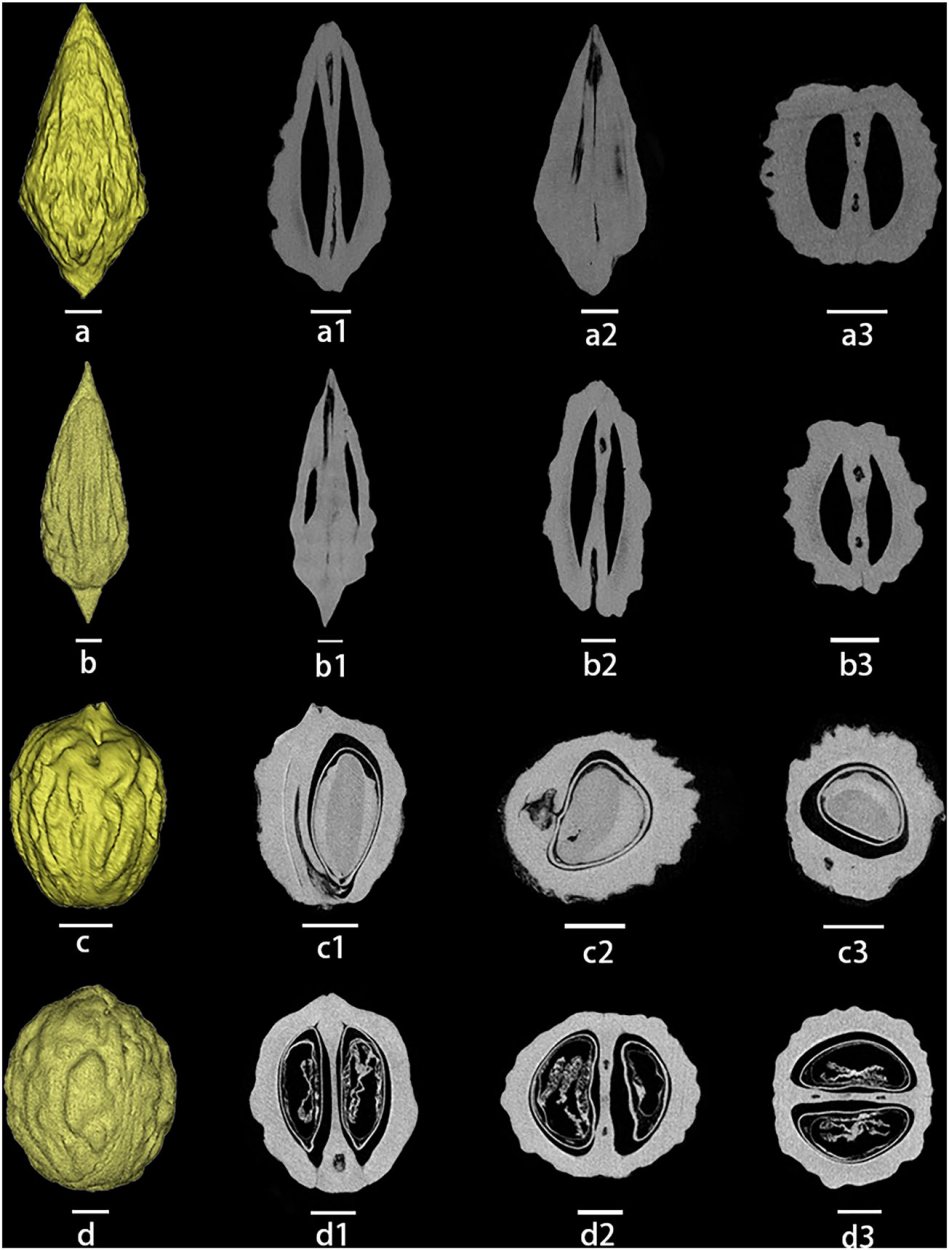
Three-dimensional structures reconstructed using CT and the internal structure of modern jujube (**a**, **b**) and sour jujube (**c**, **d**) in different views. (**a**) Zanhuangdazao; (**b**) Banzao; (**c**, **d**) Sour jujube. Scare bar = 2 mm.

CT scanning helps us a lot in visualizing the internal structure as well as the external features of the jujube kernels. The results show that one-seeded kernels were predominant among the fruit kernels excavated from the sites (Fig. 4a, e, f), but two-seeded varieties were also found in some cultivated jujubes (Fig. 4b, d). At the same time, we also performed CT scans of the kernels of modern samples and found that most modern cultivated jujubes have two seeds (Fig. 5a,b), whereas sour jujube fruits have one or two seeds in the kernel (Fig. 5c,d), a typical feature that may be related to the frequency of variation and degree of evolution of jujube trees. In addition, the seeds in the kernels of sour jujube fruits are very saturated compared with cultivated jujube fruits, which may be related to the asexual reproduction of cultivated jujube fruits. Sour jujube fruits in the wild state mainly reproduce sexually, and the saturated kernels can improve the germination rate and the survival rate, whereas cultivated jujube fruits mainly reproduce asexually, and this characteristic gradually declines in the process of cultivation over a long period of time.

## Discussion

### Evolution of kernel morphology during domestication of the jujube and sour jujube

**Figure 6:**
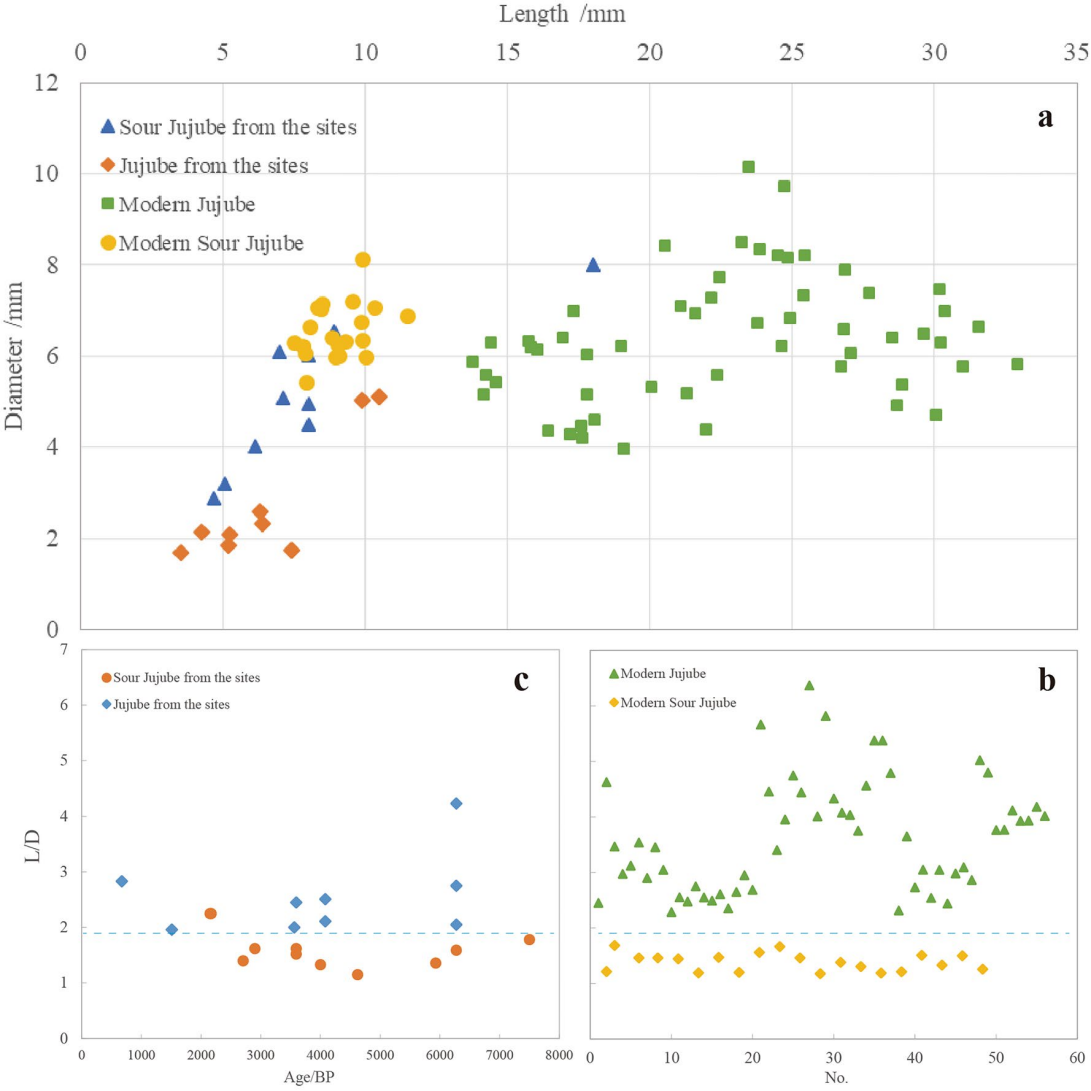
Morphometrics of jujubes and sour jujubes. (**a**) Measurements of length and diameter of jujubes and sour jujubes. (**b**) Trends in the distribution of modern kernels L/D ratios. (**c**) Trends in the distribution of kernels L/D ratios from the sites.

Kernel size and shape serve as pivotal archaeological indicators for tracing the domestication of perennial woody plant fruits^31,32^. Measurements reveal a prominent difference in length between contemporary kernel specimens of jujubes and sour jujubes, while archaeological findings at sites highlight a significant contrast in diameter (Fig. 6a). Generally, cultivated jujube kernels are smaller than those of sour jujubes, a feature potentially linked to domestication. It is reasonable to consider the possibility of parallel evolution between the jujube and sour jujube^33^. The study demonstrates that the size and shape of the jujube kernel are primarily influenced by the corresponding dimensions of the fruit. A substantial correlation exists between the overall length of the fruit and the L/D ratio of the kernel. The primary edible portion of the jujube fruit is the mesocarp. With the elongation of the kernel during domestication, the mesocarp undergoes a corresponding increase in fullness, consequently expanding the edible portion of the fruit^15,34^.

Additionally, utilizing contemporary measurements of the L/D ratios of jujube and sour jujube, we classified the kernels at the archaeological site into species (Fig. 6b, c). The results indicate a distinct distribution range of L/D values for jujube and sour jujube within the archaeological context. However, a conspicuous linear trend in their L/D values over an extended period is notably absent. This lack of trend may be ascribed to inherent biometric variations among different varieties. The kernels collected from these sites originate from diverse regions, exhibiting noticeable differences in climatic conditions, soil composition, and management strategies. Additionally, a certain lag in the exchange of production technology between different communities during the Neolithic and Bronze Age, combined with ecological isolation stemming from both natural and human factors, ultimately contributed to the parallel evolution of distinct varieties.

Significantly, the L/D value of jujube kernels found in Han Dynasty tombs is recorded as 2.25, and historical documents indicate their affiliation with sour jujube^35^. Nevertheless, in accordance with our identification criteria and the analysis of morphological characteristics, the sour jujube specimens from these tombs exhibit features indicative of either an artificially domesticated variant or a hybrid form transitioning between sour jujube and jujube. Among the kernels excavated from the three Qi River basin sites, the four cultivated jujube kernels at the Zhujia and Wangzhuang sites (Fig. 3a, f, g, i) demonstrate L/D values ranging from 2.00 to 4.23. These kernels exhibit distinct morphological disparities when compared to sour jujubes, manifesting clear signs of domestication. Upon conducting CT scans, we observed that the two jujube kernels at the Zhujia site (Fig. 4a, b) appeared to have undergone extrusion and deformation during the charring process. I Furthermore, the internal structure of these kernels lacked integrity. Based on these observations, we posit that these cultivated jujube kernels may have been softer and deformed by external forces during the burial process. In the Qi River basin, a type of non-kernel jujube, referred to as “Ruanhemizao,” is present. It possesses fine kernels and a thin endocarp, assuming a slender fusiform shape^36^. This stands in stark contrast to the noticeable differences observed in other cultivated jujube kernels. The kernels unearthed from the Zhujia site exhibit a certain degree of similarity to “Ruanhemizao,” leading us to infer that the possibility of them belonging to the same species cannot be dismissed".

### Early selection strategies for fruit trees in the Qi River Basin

Agricultural production and animal husbandry practices have a longstanding historical background in the Qi River basin of northern China, dating back to the Neolithic period^21,37,38^. During the Yangshao culture around 6000 BP, subsistence pattern based on agricultural cultivation supplemented by hunter-gathering was established^39^. Domestication and cultivation of plants added new lights into the broad spectrum of food choices and repertoire of diverse subsistence strategies. Additionally, the growing population pressure forced early communities to seek alternative food sources, making the utilization of fruit tree resources an excellent option ^5,9,40^. Notably, during this period, the domestication of the jujube entered a crucial stage in its development, significantly enriched the dietary practices of our earliest ancestors.

The intricate cultivation techniques required for the asexual propagation of fruit trees are considered the primary factor contributing to the delayed domestication of specific species ^14,15,41,42^. Wild populations typically rely on seed reproduction exclusively, and during domestication, the preservation of advantageous genes within a species becomes possible primarily through asexual reproduction. Among the earliest domestication practices, straightforward and convenient propagation techniques, such as cuttings, played a crucial role^12^. By employing methods like taking cuttings, early humans could obtain high-quality young plants, preserving specific inherited traits. Furthermore, throughout the domestication process, significant modifications can occur in the biological traits of fruit trees, encompassing changes in tree shape, leaf shape, and fruit shape. Notably, alterations in the size and shape of the fruit are inherently connected to the domestication process^43,44^.

During the initial stages of fruit tree selection, emphasis was placed on edibility; however, biological characteristics such as heightened resistance to pests and diseases, along with improved survival rates, are increasingly gaining significance in the pre-domestication cultivation phase^9,12^. In light of these considerations, jujube trees hold a distinct advantage in the expansive regions of northern China. The native variety of jujube in northern China is the sour jujube, characterized by widespread distribution and exceptional adaptability, including remarkable tolerance to drought, low soil fertility, saline soils, high temperatures, and late frosts^35,45^. These characteristics would have proven beneficial during the domestication and subsequent management by early populations. In the early selective domestication of fruit trees by humans, the specific utilization values of these trees have garnered scholarly interest^46,47^. Relevant studies have demonstrated that jujube is rich in sugar, calories, proteins, fats, and other nutritional values^48^. Furthermore, it surpasses fruits like apples, pears, and grapes in vitamin content and also exhibits certain medicinal properties^49,50^. Given this compelling evidence, the jujube tree undoubtedly stands out as one of the most worthwhile fruit trees chosen and cultivated by early communities during the Neolithic period.

### Utilization of fruit in the middle and lower Yellow River basin of northern China

The distribution of wild populations plays a crucial role in determining the origin of cultivated crops^50^. The sour jujube stands out as one of the oldest wild fruit trees in East Asia, exhibiting widespread distribution throughout northern China, particularly in the mountainous and hilly regions of Shaanxi, Shanxi, Henan, Hebei, and Shandong^35^. Fossilized leaves of the sour jujube tree discovered in the Miocene Shanwang Flora in Linqu County, Shandong Province, China, closely resemble the leaf morphology of contemporary sour jujube^51^. This significant discovery indicates that sour jujubes were extensively distributed in northern China approximately 12 million years ago, providing substantial evidence for their native origin in China^52^. The traditional geographic distribution range of cultivated jujube overlaps with that of sour jujubes, implying the widespread distribution of the latter in northern China around 12 million years ago, establishing their native origin in China^24^. Subsequently, following thousands of years of domestication in this region, jujube trees were introduced to neighboring countries such as Korea and Japan, as well as Central Asia and Europe, through the Silk Road during the Han Dynasty in China about 2000 years ago^44,53^. Therefore, the distribution pattern of jujube and sour jujube suggests that both species originated in the middle and lower reaches of the Yellow River.

Ancient Chinese historical documents explicitly record details about both jujube and sour jujube. The *Shijing*, the earliest historical document documenting the cultivation of jujube trees, contains a description of "Jujube fruit picked in August and rice harvested in October," providing evidence that jujube trees were cultivated on a significant scale as early as 3000 years ago. Additionally, the *Erya*, an ancient text, offers information on 11 distinct cultivars of jujube trees that can be traced back over 2600 years ^23^. Furthermore, the *Qi Min Yao Shu* from the Northern Wei Dynasty (1500 BP) provides comprehensive instructions on seed selection, planting techniques, and processing methods for jujube paste. Works from the Ming and Qing dynasties, including *Ben Cao Gang Mu* (Compendium of Materia Medica) and *Nong Zheng Quan Shu* (Whole book on agricultural activities), also contain valuable records related to jujube^45^.

Jujube cultivation plays a significant role in traditional agroecosystems, with a long history in China. Evidence suggests that fruit trees have been cultivated and utilized in the middle and lower reaches of the Yellow River in northern China since the middle to late Neolithic period. The reasons behind the early cultivation and utilization of fruit trees are also a topic of great interest. The domestication of perennial fruit trees is a lengthy and intricate process, and the domestication of jujube trees in the Qi River basin approximately 6,000 years ago was not a mere coincidence. Furthermore, this time period coincided with the Holocene climatic optimum^54,55^. The warm and humid climate provided an ideal natural environment for the growth of fruit trees.

Additionally, the cultivation of crops such as millet through dry farming in the region reached a stage of intensive production around 6,000 BP. This achievement was made possible due to significant advancements during the Peiligang period (9000–7000 BP) and the early Yangshao period (7000–6000 BP). Food has always played a pivotal role in the advancement of human society and the establishment of civilization^56^.It serves as a fundamental prerequisite, providing a stable source of sustenance that contributes to population growth and the development of social classes^40,57^. Population pressure in the middle and lower reaches of the Yellow River increased during the late Yangshao period. Increased food resources were necessary for sustainable development due to the substantial growth in population. Consequently, early communities made an astute choice by diversifying food sources during that time. Moreover, the domestication of the jujube tree was influenced by the stratification of social classes. As fruits were traditionally considered luxury foods and offerings to ancestors, the association between fruits and celebrations may have motivated ancient farmers to dedicate extensive labor towards cultivating and further domesticating fruits in China^40^. In this context, the process of domesticating the jujube tree in the Qi River basin started and continued extensively. It is noteworthy that agricultural surpluses facilitated the emergence of resident specialists who were liberated from food production^58^. This spurred the innovation and advancement of agricultural production technologies, providing technical assurance for the domestication and cultivation of jujube trees. In conclusion, the warm and humid climate, population pressure, and accumulation of production technologies have presented practical opportunities for jujube tree domestication in the Qi River basin.

### Conclusion

A comprehensive comparative analysis was conducted on kernel remains obtained from the Qi River basin in northern China, along with kernels from 11 distinct sites of varying periods in northern China, alongside modern specimens. This study employed a systematic morphological approach. Based on our measurements of modern fruit kernels, we identified that the extant sour jujube had L/D values ranging from 1.19 to 1.68, while the extant cultivated jujube had ratios that ranged from 2.28 to 6.37. Additionally, we observed that the L/D values of the two were distributed above and below the threshold value of 1.9, indicating a clear distribution boundary. Utilizing the observed distribution pattern, we classified the kernels on the site into different species. Our findings indicate clear distinctions in the kernel morphology of jujube and its cultivated counterpart, featuring pointed ends and elongated shapes typical of domestication. This adaptation was largely driven by the need to increase the thickness of the mesocarp, subsequently enhancing the edible portion of the fruit to meet human food demands. It is noteworthy that the domestication features of the four jujube kernels found at the Zhujia and Wangzhuang sites in the Qi River basin are prominent, and their morphological traits bear a striking resemblance to certain modern cultivated jujube varieties. This indicates that jujube cultivation began in the Qi River basin around 6200 BP at the latest. The discovery of jujube kernel remains dating back to approximately 6000 BP in central China provides compelling phytoarchaeological evidence for the domestication of jujube trees during the Neolithic period in northern China.

## Materials and methods

### Plant guidelines statement

All the samples involved in our study were collected in a legal and compliant manner, in full compliance with relevant institutional, national and international guidelines and regulations. The charred fruit kernels from the archaeological sites of Nanyangsi, Xipu, Xiwusi, Taomugang, Nansha, Zhujia, Dalaiidan, Wangzhuang and Koujia covered in this paper were collected by The Institute of Vertebrate Paleontology and Paleoanthropology (IVPP), Chinese Academy of Sciences (CAS) during previous archaeological excavations and are published for the first time. Modern sour jujube kernel specimens were obtained from The Germplasm Bank of Wild Species (Kunming, Yunnan Province, China), and cultivated jujube kernels were collected by IVPP in the core areas of different cultivated jujube species.

### Archeological sample collection

In June 2021, we cooperated with The Palace Museum and the Henan Provincial Institute of Cultural Heritage and Archaeology to collect samples from three sites: Dalaidian, Zhujia, and Wangzhuang (Supplementary Fig. S1). In addition, we collected charred kernels from six sites in the middle and lower reaches of the Yellow River in northern China, including Nanyangsi, Xipo, Xiwusi, Taomugang, Nansha and Koujia, as supporting materials for this study, and the relevant samples are preserved in the Key Laboratory of Vertebrate Evolution and Human Origins of the Chinese Academy of Sciences. The samples were obtained primarily from ash pits that were buried within the strata or from sections that contained cultural layers. To prepare the samples for analysis, we used a flotation technique to separate the organic material. Subsequently, we collected the results of the flotation, dried them in a cool and well-ventilated area, and transported them back to the Key Laboratory of Vertebrate Evolution and Human Origins at the Institute of Vertebrate Paleontology and Paleoanthropology, Chinese Academy of Sciences. At the laboratory, the samples were carefully sorted, classified, identified, photographed, and analyzed using a Leica M205 C stereo microscope and LAS V4.12 software. The identified phytoarchaeological remains can be broadly categorized into three types, namely charred cereal seeds, weed seeds, and kernels. Among these, charred cereal seeds are the most dominant. Fruit kernels are less frequently encountered and comprise only a small fraction of the excavated plant remains.

### Chronology

A total of four AMS dating samples were selected from the Dalaidian, Wangzhuang, and Zhujia sites, of which one charred millet seed and one charcoal sample were selected from the Zhujia site in T3-1 and T2-6, respectively. A charcoal sample was selected from the Dalaidian site at a depth of 270 cm in Sections 1–1. A charcoal sample from the ash pit 2–4 was selected from the Wangzhuang site. The samples used for AMS^14^C dating were completed on the Beta Lab, and the dating results were corrected according to the Intcal20 calibration curve using the software Calib 8.20 (http://calib.org).

### Modern sample

The middle and lower Yellow River basin in northern China is widely recognized as the region where jujube originated earliest and underwent domestication. In order to thoroughly study the morphological changes of jujube during domestication and cultivation, we collected the six most common cultivars and three sour jujube varieties from Gansu, Shaanxi, Shanxi, Henan, Hebei, Shandong, and Inner Mongolia provinces (Supplementary Figs. S2, S3). Subsequently, we measured the morphology of their kernels. Specifically, ten kernels were randomly selected from each variety and their mean value was measured.

### Morphological analysis

Morphometric methods have been extensively utilized in the analysis of cereals, legumes, and fruits to examine population variations, and their validity has been widely confirmed^59,60^. Jujube and sour jujube kernels present distinct morphological differences, which can be accurately assessed through simple morphometric measurements. Consequently, we conducted a comprehensive analysis of jujube kernels excavated from archaeological sites located in the middle and lower regions of the Yellow River in northern China. This analysis primarily involved measurements of kernel length and diameter (Supplementary Table S2).

### Image acquisition

Computed Tomography (CT) is an ideal non-destructive technique for studying the internal structure of samples, allowing the acquisition of thousands of continuous images that can be used to produce accurate reconstructions of internal morphology^61^. To observe the internal structure of date palm kernels more clearly, we performed 225kV three-dimensional fossil micro-CT (225-3D-μCT) scans on some date palm kernels. The raw projections were converted to tomographic slices in raw format using IVPP225kVCT_Recon software, then the raw tomograms were imported into VGstudio 2.2 software and saved. Tiff files, and finally imported into Mimics 19.0 software to obtain images of the 3D structures and different views.

### Supplementary Information

Supplementary Figures.

Supplementary Table S1.

Supplementary Table S2.

## Data Availability

The datasets used and analysed during the current study available from the corresponding author on reasonable request.
