# Author Correction: Cultivation and morphology of jujube (*Ziziphus Jujuba* Mill.) in the Qi River Basin of Northern China during the Neolithic Period

**DOI:** 10.1038/s41598-024-60573-x

**Published:** 2024-05-02

**Authors:** Yanpeng Li, Xinying Zhou, Keliang Zhao, Junchi Liu, Guanhan Chen, Yaping Zhang, Jiacheng Ma, Nan Sun, Xiaoqiang Li

**Affiliations:** 1 School of Earth Science and Resources, Chang’an University, Xi’an, 710054 China; 2 Key Laboratory of Vertebrate Evolution and Human Origin of Chinese Academy of Sciences, Institute of Vertebrate Paleontology and Paleoanthropology, Chinese Academy of Sciences, Beijing, 100044 China; 3 CAS Center for Excellence in Life and Paleoenvironment, Beijing, 100044 China; 4 University of the Chinese Academy of Sciences, Beijing, 100049 China

Correction to: *Scientific Reports* 10.1038/s41598-024-52260-8, published online 27 January 2024

The original version of this Article contained an error in the Acknowledgements section.

“The financial support include National Key Research and Development Program of China (2022YFF0801502), the CAS Project for Young Scientists in Basic Research (YSBR-019), and the National Natural Science Foundation of China (NSFC 41888101), and the Youth Innovation Promotion Association of the Chinese Academy of Sciences (2022071).”

now reads:

“The financial support include National Key Research and Development Program of China (2022YFF0801102), the CAS Project for Young Scientists in Basic Research (YSBR-019), and the National Natural Science Foundation of China (NSFC 41888101), and the Youth Innovation Promotion Association of the Chinese Academy of Sciences (2022071).”

The original Article has been corrected.

