# Supplementary material for: Cultivation and morphology of jujube (Ziziphus Jujuba Mill.) in the Qi River Basin of Northern China during the Neolithic Period
Source: Sci Rep. 2024 Jan 27;14:2305. doi: 10.1038/s41598-024-52260-8 (PMC10821880; doi:10.1038/s41598-024-52260-8)
Supplement: Supplementary file 1 — Supplementary Figures. [file 41598_2024_52260_MOESM1_ESM.docx]

Cultivation and Morphology of Jujube (*Ziziphus Jujuba* Mill.) in the Qi River Basin of Northern China during the Neolithic Period

Yanpeng Li^1,2^, Xinying Zhou^2,3,4, *^, Keliang Zhao^2,3,4^, Junchi Liu^2,3,4^, Guanhan Chen^2,3,4^, Yaping Zhang^2,3,4^, Jiacheng Ma^2,3,4^, Nan Sun^1, *^& Xiaoqiang Li^2,3,4^

1 School of Earth Science and Resources, Chang’an University, Xi’an 710054, China;

2 Key Laboratory of Vertebrate Evolution and Human Origin of Chinese Academy of Sciences, Institute of Vertebrate Paleontology and Paleoanthropology, Chinese Academy of Sciences, Beijing 100044, China;

3 CAS Center for Excellence in Life and Paleoenvironment, Beijing 100044, China;

4 University of the Chinese Academy of Sciences, Beijing 100049, China

* Correspondence: [zhouxinying@ivpp.ac.cn](mailto:zhouxinying@ivpp.ac.cn(Zhou); [sunnan@chd.edu.cn](mailto:sunnan@chd.edu.cn)


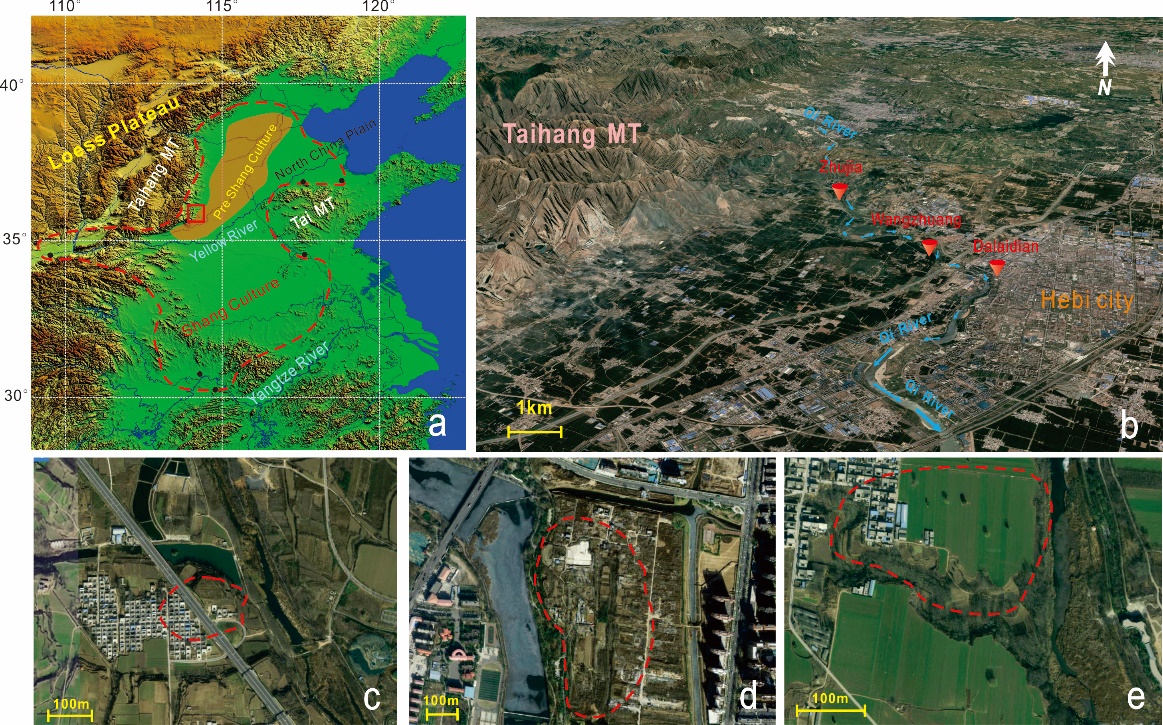


**Figure S1.** Research area and Archeological site. (a) The geographic location of the study area and distribution of cultural areas. (b) Overview of the Qi River Basin. (c) Zhujia site. (d) Dalaidian site. (e) Wangzhuang site. This map is created using ArcGIS v10.6 (<https://www.esri.com/>), in-map labels were added in CorelDraw X8 v18.1.0.690 (<https://www.coreldraw.com/>).

The Qi River basin is located in the transition zone between the eastern foothills of the Taihang Mountains and the North China Plain, with decreasing elevation from west to east and large undulations (Fig.S1). The topography of the basin is complex, with 86% of the area being hilly and mountainous, forming a typical vertical belt spectrum of the mountains. The vertical band spectrum of vegetation in the southern section of Taihang Mountains from bottom to top is scrub and crop belt, deciduous broad-leaved forest belt, mixed coniferous and broad-leaved forest belt, and scrub meadow belt. The region has a warm temperate semi-humid monsoon climate with four distinct seasons, abundant light, and large temperature differences. Spring is windy and rainy, summer is hot and humid, autumn is high and cool, and winter is cold and foggy. The average annual temperature is 14.2-15.5℃, annual precipitation is 349.2-970.1mm, annual sunshine hours are 1787.2-2566.7h (<http://data.cma.cn/>), precipitation is mainly concentrated in summer and autumn, and the four seasons are distinct, with significant non-cyclical changes in weather.

This study mainly includes three sites in the Qi River basin, Zhujia, Dalaidian, and Wangzhuang. The Dalaidian site is located on a terrace on the north bank of the Qi River at the western edge of Hebi City, covering an area of about 300,000 square meters. The site is large and rich, with cultural deposits from different periods, including Yangshao, Longshan, Xia Dynasty and Shang Dynasty, especially during the Longshan, Shang, and Zhou dynasties when the area became a large central settlement. Wangzhuang site is a relatively well-preserved small settlement site, located 2km northwest of the Dalaidian site, archaeologists excavated the site for the first time in the first half of 2014, the cultural accumulation includes different periods such as Yangshao, Longshan, and pre-Shang, but the pre-Shang culture is the main. The Zhujia site is located on the east side of Zhujia village in the northwest of Hebi city and is a Neolithic site.


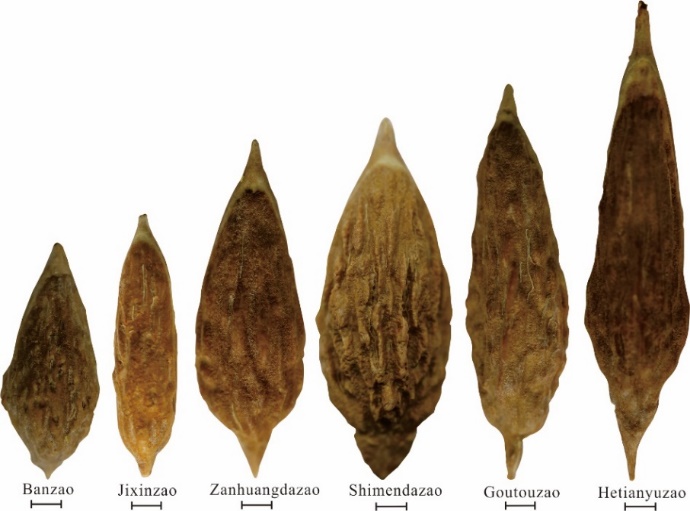
**Figure S2.** The kernels of modern jujube (*Ziziphus jujuba* Mill.) Scale bar=2 mm

We collected modern jujube kernels from various regions of northern China. The morphology of the kernels exhibits significant variation among different varieties, yet some common features persist, such as the presence of obvious small tips at both ends—a distinctive characteristic of cultivated jujube (Fig. S2). Specifically, Banzao is sourced from the south of Shanxi Province, Jixinzao is gathered in the western part of Henan Province, Zanhuangsdazao is picked up in the western part of Hebei Province, Shimendazao is harvested in the central part of Gansu Province, Goutouzao is collected in the northern part of Shaanxi Province, and Hetianyuzao is obtained from the eastern part of Xinjiang Province.


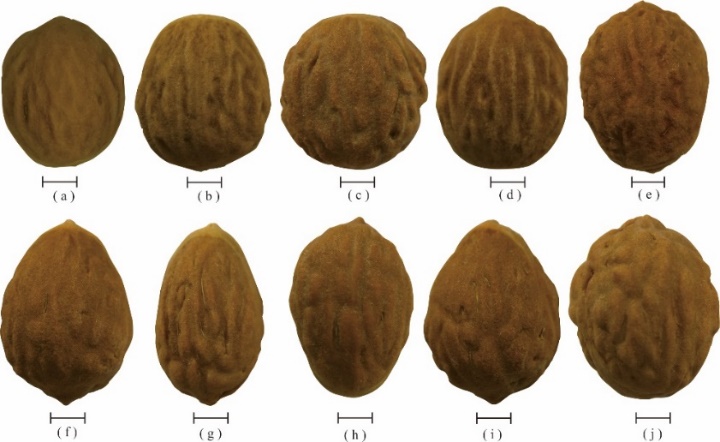


**Figure S3.** The kernels of modern sour jujube (*Ziziphus jujuba* var. *spinosa*) Scale bar=2 mm

We collected morphological abundance of modern sour jujubes, but the morphological characteristics of the pome are not very obvious relative to cultivated jujubes. Among them, (Fig. S3 a-c) are from Muping County, Shandong Province, (Fig. S3 d, e, h) are from Xingtai, Hebei Province, and (Fig. S3 f, g, i, j) are from Xincheng, Inner Mongolia. Modern sour jujube specimens were purchased from The Germplasm Bank of Wild Species (http://www.genobank.org/), order no. 23021509262229.
